# Manual Dexterity Abilities and Dual Tasking in Children With Developmental Coordination Disorder and Typically Developing Children

**DOI:** 10.1002/jclp.70051

**Published:** 2025-10-21

**Authors:** Eleonora Bieber, Bouwien Smits‐Engelsman, Giuseppina Sgandurra, Giada Martini, Anna Basu, Andrea Guzzetta, Giovanni Cioni, Hilde Feys, Katrijn Klingels

**Affiliations:** ^1^ Department of Developmental Neuroscience IRCCS Fondazione Stella Maris Pisa Italy; ^2^ Faculty of Health Sciences, Physical Activity, Sport and Recreation (PhASRec) Potchefstroom South Africa; ^3^ Department of Clinical and Experimental Medicine University of Pisa Pisa Italy; ^4^ Department of Paediatric Neurology Population Health Sciences Institute, Great North Children's Hospital Newcastle upon Tyne UK; ^5^ Department of Rehabilitation Sciences KU Leuven Leuven Belgium; ^6^ REVAL Rehabilitation Research Center, Faculty of Rehabilitation Sciences Hasselt University Diepenbeek Belgium

**Keywords:** assessment, cognitive functioning, neuroscience

## Abstract

Poor manual skills in children with Developmental Coordination Disorder (DCD) may be dependent on task complexity and due to difficulties in the automatization phase of the motor learning process. Increased task demands and the dual‐task paradigm can be used to test these hypotheses.

**Objectives:**

We aim to investigate (1) manual dexterity abilities using increased levels of difficulty; (2) dual tasking using an experimental protocol of the Tyneside Pegboard Test (TPT).

**Method:**

Sixteen children with DCD and 16 age‐matched typically developing (TD) children were included. Various experimental conditions of the TPT (unimanual, bimanual and dual task) were administered. The dual‐task paradigm comprised a primary unimanual task and a cognitive task (auditory non‐verbal task). Parents were asked to fill out the eConners questionnaire to report attentional difficulties. Repeated measures ANOVAs were used to compare possible differences in effects on the performance of the groups. Pearson correlation coefficients were calculated between dual‐task performance and ADHD index of the eConners questionnaire.

**Results:**

Children with DCD performed significantly worse in all task conditions (unimanual, bimanual, dual task) compared to TD children. In unimanual and bimanual conditions, they did no not present a higher impact of task constraints. Dual‐task performances did not have a differential effect on groups and were not interfered by attentional difficulties.

**Conclusions:**

Children with DCD exhibit a general slowness in all TPT tasks. Our findings do not support the automatization deficit hypothesis.

## 1 Introduction

Developmental Coordination Disorder (DCD) is a neurodevelopmental condition affecting approximately 5%–6% of school‐aged children (Smits‐Engelsman et al. 2015).

According to the DSM‐5, DCD is defined by four criteria: (1) significant difficulties in acquiring and executing motor skills, (2) interference with daily functioning, (3) early onset, and (4) exclusion of other neurological or intellectual disorders (American Psychiatric Association 2013).

These children exhibit deficits in performing motor tasks appropriate for their age. Their movement difficulties, particularly in fine motor performance, hinder their academic progress or their accomplishment of activities of daily living (ADL) (Smits‐Engelsman and Verbecque 2022).

### 1.1 Manual Dexterity Abilities in Children With DCD

Slow and clumsy execution of fine motor tasks is frequently observed in children with DCD and is referred to as a manual dexterity deficit. Difficulties in fine motor skills, such as in‐hand manipulation, bimanual coordination, target‐directed reaching, and handwriting, have been widely reported (Feder and Majnemer 2007; Huau et al. 2015). Several tests have been used to assess these components of manual dexterity in children with DCD (Bieber et al. 2016), typically by evaluating speed and accuracy. One of the most commonly used assessments is the Movement Assessment Battery for Children (mABC‐2) (Henderson et al. 2007). The mABC‐2 test includes tasks performed with both the dominant and nondominant hands. However, normative scoring is based on combined performance across the manual dexterity subtests and does not provide separate interpretation by hand. For this reason, our study chose to examine hand‐specific performance to gain further insight into the potential role of hand dominance in manual dexterity.

In a large sample of children with and without DCD, aged 5–6 years, Asonitou and colleagues (2012) evaluated the manual dexterity domain of the mABC‐2 and found significant differences in unimanual and bimanual speed tasks between the two groups. Similarly, Farhat and colleagues (2016) reported significant differences across all manual dexterity tasks in the mABC‐2 test. Also pegboard tests provide valuable insights into the speed and accuracy of manual dexterity in children with DCD. Commonly used pegboard assessments include the Purdue Pegboard Test (Pitcher et al. 2003; Wilson et al. 1982), the Tyneside Pegboard Test (Basu et al. 2018), the Nine‐Hole Peg Test from the NIH Toolbox (Reuben et al. 2013; Wang et al. 2011), and the Grooved Pegboard Test (Kanj et al. 2022). Kanj and colleagues (2022) reported significant differences in manual dexterity between children with and without DCD using the Grooved Pegboard Test (GPT), but only for the dominant hand. However, since the sample of children with DCD was small (*n* = 8), these findings should be interpreted with caution.

The Tyneside Pegboard Test (TPT) (Basu et al. 2018) is a quantitative tool developed specifically to assess uni‐ and bimanual dexterity abilities in children with cerebral palsy, but can also be used in other pediatric populations, such as children with DCD.

Although there is an abundance of evidence that children with DCD perform poorly in manual dexterity tasks, less is known about the impact of specific task constraints on task speed and whether results would differ between dominant and nondominant hands.

### 1.2 Automatization Deficit Hypothesis and Dual‐Task Paradigm in Children With DCD

The ability to perform tasks with minimal cognitive effort—referred to as automaticity—is a key feature of skilled motor performance (Stefanidis et al. 2007). As individuals acquire motor proficiency, their reliance on attentional resources diminishes, enabling more efficient execution and allowing for multitasking. However, when two tasks are performed concurrently, cognitive‐motor interference (CMI) may arise, leading to performance decrements in one or both domains (Schott et al. 2016). This interference becomes particularly evident when the tasks are not fully automated and thus demand higher cognitive input.

Children with DCD often experience significant difficulties acquiring and executing motor skills. The automatization deficit hypothesis suggests that these children struggle to automate movements and require greater attentional resources than their typically developing (TD) peers (Visser 2003). This reduced capacity for automaticity may increase their susceptibility to dual‐task interference and contribute to their functional limitations in everyday activities.

To explore the relationship between cognitive load and motor performance, the dual‐task paradigm offers a well‐established method. This paradigm requires participants to perform two tasks simultaneously—either combining cognitive and motor elements or involving two motor tasks—allowing researchers to assess how shared attentional resources are allocated (Navon and Gopher 1979). The paradigm is ecologically valid, mirroring everyday multitasking scenarios such as walking while conversing (Pena et al. 2019).

A particularly relevant variant, the cognitive‐motor dual‐task (CMDT), involves pairing a motor task (e.g., walking, balancing) with a cognitive task (e.g., working memory, attention, or decision‐making), and has been extensively used in DCD research (McIsaac et al. 2015; Bayot et al. 2018). Most studies to date have focused on gross motor tasks, typically using gait or postural control as the motor component and simple verbal or sequencing tasks as the cognitive load (Cherng et al. 2009; Laufer et al. 2008; Schott et al. 2016). Results, however, have been inconsistent: some show greater dual‐task costs in children with DCD (Chen et al. 2012; Tsai et al. 2009), while others find comparable performance between DCD and TD groups (Biotteau et al. 2015; Jelsma et al. 2021; Krajenbrink et al. 2023). These discrepancies may be due to variations in task design, complexity, or the nature of the secondary task (Schott et al. 2016).

To address such variability, Wollesen et al. (2019) proposed a task modality taxonomy that classifies dual‐task conditions by sensory and cognitive demands. For example, auditory non‐verbal tasks (e.g., tone or rhythm discrimination) are distinct from verbal tasks, which require linguistic processing. Esmaeili Bijarsari (2021) expanded this framework by incorporating task complexity and specifying cognitive domains such as executive control, attention, and working memory—providing a more comprehensive model for interpreting dual‐task performance.

Recently, attention has shifted toward fine motor tasks in dual‐task contexts, recognizing their importance for activities of daily living (ADL) and the frequent fine motor difficulties observed in children with DCD. For instance, Krajenbrink et al. (2023) paired a manual dexterity task with either a concurrent motor or cognitive task. Although overall dual‐task costs did not significantly differ between DCD and TD groups, children with DCD exhibited greater mental effort and more pronounced performance declines when the tasks involved complex fine motor and motor coordination components.

In the present study, we extend this line of inquiry by using a fine motor task as the primary activity and an auditory non‐verbal task as the secondary load. This combination was deliberately chosen to avoid confounding variables associated with visual processing, which is frequently impaired in children with DCD (Pinero‐Pinto et al. 2022); and with verbal tasks, which may be problematic due to the high comorbidity of DCD with language disorders and executive function deficits (Leonard and Hill 2014; Fogel et al. 2021).

This methodological design allows for a more precise assessment of cognitive‐motor interference in fine motor execution, enhancing our understanding of automatization challenges in children with and without DCD.

### 1.3 Objectives of the Study

Based on this theoretical framework and methodological rationale, the present study firstly aims to:to gain insights into unimanual and bimanual dexterity abilities in a sample of children with DCD, compared with age‐matched TD children, using the TPT, with manipulation of different task constraints such as size of peg (small vs large), movement direction (nondominant to dominant side), hand used (dominant vs nondominant), number of hands used (uni‐ vs bimanual), on the time needed to complete the task. Secondly, we aimed to investigate the automatization deficit hypothesis through the paradigm of a dual motor ‐ auditory non‐verbal task, and the influence of attentional difficulties on dual‐task performance. Based on reported findings, we hypothesized that a) children with DCD would present with lower speeds in the performance of manual tasks than TD children, and that b) the influence of increased task constraints would be greater in children with DCD, such that; smaller pegs will lead to longer completion times than larger pegs; movements from the nondominant to the dominant side will take more time than the reverse; tasks performed with the nondominant hand will result in poorer performance than those with the dominant hand; bimanual tasks, which place higher demands on motor coordination, will require more time than unimanual tasks. We also hypothesized that, c) in the dual‐task condition, children with DCD show decreased motor performance leading to slower movement, especially with the nondominant hand. Furthermore, d) since attentional demands are higher in a dual task, attentional problems would further impair dual task performance. To achieve these goals, we developed a specific protocol to investigate manual dexterity in children with DCD and TD children through the execution of unimanual, bimanual, and dual tasks using the TPT.

## 2 Materials and Methods

### 2.1 Participants

Children with DCD aged 6–10 years were recruited between February 2018 and October 2021 at the Stella Maris Scientific Institute (Pisa, Italy). Age‐matched TD children were recruited via colleagues, friends, and families of the investigators between May 2019 and April 2022. This study was approved by the Ethical Committee (43/2017) of the Department of Developmental Neuroscience of IRCCS Stella Maris.

All parents provided written informed consent for their children's participation. Children aged 7 years and older provided written consent. For children under the age of 7 verbal assent was provided after giving them verbal information on the testing using age‐appropriate language. Participants with DCD met all four diagnostic criteria outlined in the DSM‐5 (American Psychiatric Association 2013): a) For Criterion A, motor impairment was assessed using the Movement Assessment Battery for Children—Second Edition (mABC‐2; Henderson et al. 2007), with a total score at or below the 16th percentile according to the Italian validation (Zoia et al. 2019); b) for Criterion B, motor skill deficit significantly and persistently interferes with ADL as evidenced by the Developmental Coordination Disorder Questionnaire (DCDQ’07) completed by parents (Parmar et al. 2014);c) for Criterion C, onset of symptoms in the early development period, as confirmed by interview with parents; d) for Criterion D, absence of any medical condition that could cause the motor impairment and IQ ≥ 70, as evidenced by psychological assessment (Wechsler Preschool and Primary Scale of Intelligence (WPPSI) (Wechsler 2012); Wechsler Intelligence Scale for Children (WISC III, IV) (Wechsler 1991, 2003), and a clinical neurological assessment performed by a paediatric neurologist.

TD children were included if they had (1) scores above 25th percentile on the mABC‐2; (2) a total score in the area of “probably no DCD” at the DCDQ’07 questionnaire (Parmar et al. 2014) (3) spoke and understood Italian. Cut‐off scores for suspected DCD on the DCDQ'07 were 15–46 for children aged 5.0–7.11 years, 15–55 for children aged 8.0–9.11 years, and 15–57 for children aged 10.0–15 years. TD Children were excluded if they had intellectual disabilities or neurological conditions, as confirmed by anamnesis of the parents.

### 2.2 Assessments and Procedure

All children were assessed individually in a quiet room during a single session lasting approximately 40 min. They first performed the mABC‐2 test (Henderson et al. 2007), followed by the TPT (Basu et al. 2018) (unimanual, bimanual and dual‐tasks conditions). During each child's evaluation, parents were asked to complete the short form of the Conners’ Parents Rating Scale (CPRS‐R:S) (Conners 2001) and the DCDQ’07 (Parmar et al. 2014).

#### 2.2.1 Outcome Measures

##### 2.2.1.1 Motor Task

The TPT (Basu et al. 2018) was used to measure uni‐ and bimanual dexterity. Unimanual tasks comprise moving nine pegs (small, medium and large) from one board to an adjacent board as quickly as possible, using dominant and nondominant hands. In the bimanual task, pegs are picked up one by one with one hand, passed through a hole in a perspex screen to the other hand, and placed in the adjacent board.

**Figure 1:**
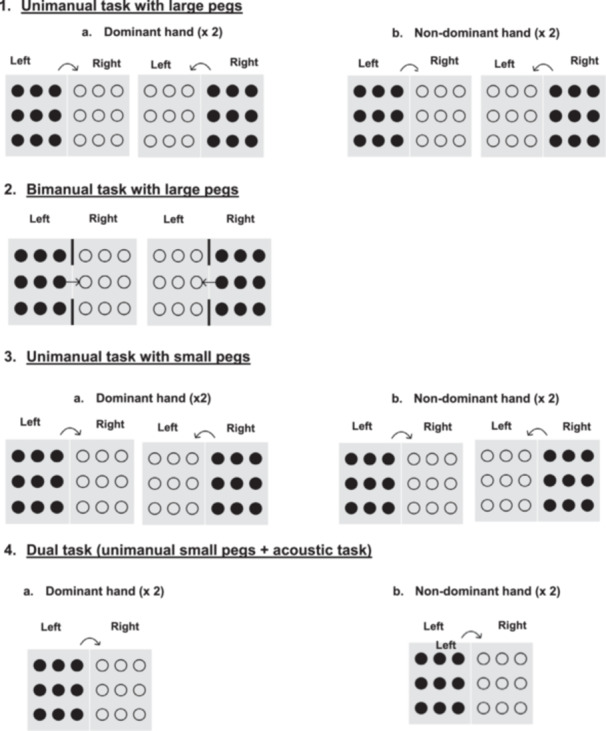
Overview of unimanual and bimanual Tyneside Pegboard Test tasks.

Although the TPT is an electronic device which enables completion times to be collected through custom‐written software, in our study, we did not collect the data electronically because we had designed a specific protocol (see Figure 1) in accordance with our study aims, which did not follow the procedure of the software. Times were recorded with a digital stopwatch.

A lower score on the TPT represents a faster and better performance. In participants with normal development, from 4 to 80 years old, test‐retest reliability (intraclass correlation coefficient 0.74–0.91), and concurrent validity with the Purdue Pegboard test (*r* = 0.61) have been established. While both the TPT and the Purdue Pegboard Test are designed to assess manual dexterity through unimanual and bimanual tasks, they differ notably in terms of apparatus design, administration procedures, and scoring methods. The Purdue Pegboard Test employs a rectangular board equipped with small metal pegs, two vertical columns of 25 holes, and four concave cups at the top. Participants are required to insert as many pegs as possible into the holes within a 30 s time frame, using the dominant hand, nondominant hand, and both hands simultaneously across three trials. Scoring is based on the number of individual pegs or peg pairs placed, with average performance calculated over the trials (Buddenberg and Davis 2000).

Construct validity in children with unilateral cerebral palsy has been reported with Assisting Hand Assessment (*r* = 0.63–0.69) and ABILHAND (*r* = 0.62–0.65) (Basu et al. 2018). No construct validity of the TPT is reported in children with DCD.

##### 2.2.1.2 Dual Task

The dual‐task condition combined the unimanual motor task (small pegs, left‐to‐right direction) with a simultaneous auditory non‐verbal discrimination task. Participants were instructed to verbally respond “yes” upon hearing the sound of a helicopter, distinguishing it from an airplane. The sounds were presented in a randomized sequence via an MP3 audio file on a personal computer.

Before performing the dual‐task condition, participants completed practice trials that included sound recognition as well as motor trials using three pegs. These practice sessions ensured that they were familiar with each component of the dual‐task before proceeding to the actual testing phase. The best time of the two trials for each unimanual condition was used for data analysis.

##### 2.2.1.3 Protocol Procedure

Figure 1 provides a graphical representation of the protocol conditions. The complete set of task variations—designed to ensure a randomized administration order—is detailed in Appendix A. A total of six protocol variations were constructed to randomize the order of block presentation and task parameters across participants, ensuring a counterbalanced design

Each block varied in starting hand (dominant vs. nondominant) and direction of movement (left‐to‐right or right‐to‐left), based on the participant's handedness, which was determined by observing the hand used for writing. For right‐handed participants, left‐to‐right corresponds to nondominant‐to‐dominant movement; for left‐handed participants, the reverse applies.

The left‐to‐right direction was selected as the reference condition, following Basu et al. (2018), as it reflects the natural direction used in writing and other everyday manual activities. In unimanual tasks with both small and large pegs, participants completed trials in both directions. They were verbally instructed to move the pegs as quickly as possible using the hand specified in the protocol. Each unimanual condition was repeated twice, and the faster execution time was recorded for analysis, in line with mABC‐2 procedures.

In the bimanual task, participants passed a peg from one board to another through a hole in a Perspex screen, completing one trial from left to right and one from right to left.

The dual‐task condition combined the manual task with an auditory non‐verbal task and was administered only in the left‐to‐right direction using small pegs, to increase task difficulty and sensitivity to automatization deficits.

For comparisons between single‐ and dual‐task performance, only trials performed in the reference direction (left‐to‐right) were included—corresponding to nondominant‐to‐dominant movements for right‐handed children and dominant‐to‐nondominant for left‐handed children.

#### 2.2.2 Diagnostic Measures

##### 2.2.2.1 Motor Performance Measure

*Motor problems* in children with DCD were assessed using the Italian version of the mABC‐2 (Zoia et al. 2019). Normative data are available for children aged 3 to 16 years old in three different age bands (3–6 y, 7–10 y, 11–16 y). Validity and reliability of the mABC‐2 test have been recently re‐examined in a sample of 273 children with and without DCD, aged 4 to 9 years, revealing a range of good to excellent internal consistency (Cronbach's α range 0.59–0.91). Moderate concurrent validity has been reported between mABC‐2 and the DCDQ’07 (*r* = 0.60, *p* < 0.001) and between the mABC‐2 test and the Go/No‐Go test (St. St John et al. 2019) (*r* = 0.50, *p* < 0.001) (Ghayour Najafabadi et al. 2022).

##### 2.2.2.2 ADL Measure

*ADL* were assessed through parents’ completion of the Italian *DCDQ’07* (Parmar et al. 2014). This is a screening tool specific for children with DCD, providing an indication of children's everyday motor functioning at home, at play and at school. According to three age bands (5 years to 7 years 11 months; 8 years to 9 years 11 months; 10 to 15 years), the score indicates if a child is “suspected of DCD” or “probably [has] no DCD.” Cross‐cultural validation and reliability (internal consistency) of the Italian DCDQ’07 was *α* = 0.94 for total score. Item‐total correlation ranged from 0.58 to 0.82. Test‐retest reliability indices were high, ranging from 0.82 to 1. Good predictive validity was also established (Caravale et al. 2014).

##### 2.2.2.3 Attention Measure

The short form of the Italian version for parents of the Conners’ Rating Scale (CTRS‐R:S) (Conners 2001) was used to investigate the presence of symptoms relevant to *attention deficit hyperactivity disorder (ADHD) and related disorders*. This questionnaire was developed for children and adolescents aged 6–18 years and includes 28 items divided into four content subscales: oppositional, cognitive problems/inattention, hyperactivity, and the ADHD Index which assesses the core symptoms of ADHD (i.e., inattention and hyperactivity/impulsivity). For each subtest, T scores are interpreted as follows: 70+ : very elevated score, indicating many more concerns than typical; 65–69: elevated score, indicating more concerns than typical; 60–64: high average score, indicating slightly more concerns than typical; 40–59: average score, indicating typical levels of concern; < 40: low score, indicating fewer concerns than typical. The ADHD index scores were considered for the data analysis. Normative data are available for each scale according to sex. Excellent internal reliability has been reported (Conners et al. 1998) ranging from 0.73 to 0.95 for males and from 0.76 to 0.94 for females, in TD children aged 3 to 17 years. Known‐group validity was established in children with ADHD and TD (Conners et al. 1998). Questionnaires on attention and on ADL were given to the parents before the start of the motor assessment.

### 2.3 Statistical Analysis

Preliminary analyses were performed to ensure no violation of the assumptions of normality, linearity and homoscedasticity. These were based on generating a scatterplot to check for outliers, examining the distribution of the data points and determining the direction of the relationship between the variables.

Baseline differences in demographic and clinical characteristics were investigated using the chi‐squared test (sex) and Mann‐Whitney U Test (age, mABC‐2 total score and manual dexterity domain, DCDQ'07, eConner subscales). As the Shapiro‐Wilk tests showed normal distribution of the TPT parameters, parametric tests were used.

We first examined if children with DCD and TD children differed in their performance on the different TPT unimanual conditions, using a repeated measures ANOVA with a factorial design. The analysis comprised 2 × 2 × 2 analyses, with size of pegs (large vs small), hand used (dominant vs nondominant hand), and direction of the task (from nondominant to dominant side and vice versa) as within‐subject variables, and group (DCD vs TD) as between subject variable. Secondly, a repeated measures ANOVA was conducted to compare scores on unimanual (dominant and nondominant hand) and bimanual tasks. Thirdly, we investigated group differences between the single task and dual‐task with a repeated measures ANOVA, with task (single vs dual) and hand (dominant and nondominant hand) as within‐subject variables, and group (DCD vs TD) and as between‐subject variable.

Finally, we investigated the relationship of the outcomes of dual‐task performance (dominant and nondominant hand) and attentional difficulties, as measured by the ADHD index of the eConners questionnaire. A partial correlation was conducted, controlling for group, to account for potential confounding effects of diagnostic status and isolate the association between attentional scores and motor performance. Bonferroni post‐hoc analysis was conducted to examine differences between groups. Effect sizes with partial eta squared were interpreted as small (0.2), medium (0.5) and large (0.8) (Cohen 2013). A significance level of *p* < 0.05 was set. All analyses were performed using SPSS Version 25 (IBM Corp. 2017).

## 3 Results

### 3.1 Participants

Table 1 displays demographic and clinical characteristics of the participants. Twenty children with DCD were enrolled in the study. Four children were excluded because they did not manage to perform all the TPT tasks for behavioural reasons such as lack of collaboration and/or severe attentional difficulties. In total, the data of 16 children with DCD (mean age 7y9m, SD 1y2m, 4 F:12 M) were analysed. The mABC‐2 percentile scores of the children ranged between 0.1 and 9. Twenty‐five TD children were tested. Nine TD children were excluded because they presented a poor motor profile (mABC‐2 total percentile score below 25). The data of 16 individually age matched TD children (mean age 7y9m, SD 1y4m; 7 F:9 M) were included in the analysis. The mABC‐2 percentile scores ranged between 25 and 98. No significant difference was found between groups for age (*p* = 0.33) and gender (*p* = 0.24). The DCD group had significantly poorer motor performance than the control group, both on total scores and in the manual dexterity subdomain of the mABC‐2 test (*p* < 0.001). DCD children scored significantly lower than controls on the DCDQ’07 questionnaire (*p* < 0.001). All TD children presented average values in the subscales of the eConner questionnaire. Children with DCD presented vey elevated score in the subscale of cognitive problems/inattention (mean 74.1, SD ± 14.9) and in the one of ADHD's index (mean 73.2, SD ± 15.07). Vey elevated scores were also reported in the subscale of oppositivity (mean 60.3, SD ± 15.4) and of hyperactivity (mean 66.0 ± 16.0). Table 1a shows how many children in each group fell within the normative range for each subscale of the eConners questionnaire. A significant difference between groups was found in all the subscales of the eConner questionnaire.

**Table 1:** Participants’ demographics, clinical characteristics, and statistics of differences between groups.

	DCD	TD	*p*
*N*	16	16	
Gender (Male:Female)	12:4	9:7	0.24
Age (mean years ± SD)	7y9m ± 1y2m	7y9m ± 1y4m	0.65
IQ (mean standard score ± SD)	99.9 ± 14	—	
mABC‐2 Median percentile (IQR)	5 (5–5)	56.5 (46.8–75)	< 0.001
mABC‐2 (Manual dexterity) Median percentile (IQR)	5 (2–9)	37 (9–68.3)	< 0.001
DCDQ’07 Median total score (IQR)	39 (35–42.5)	64.5 (60.25–71)	< 0.001
ADHD index (Median, IQR)	(*N* = 15) 78.0 (61.5–84.5)	(*N* = 11) 44.0 (40–51)	< 0.001
Hyperactivity (Median, IQR)	(*N* = 15) 68.0 (53.3–76.8)	(*N* = 11) 42.0 (39.5–52)	< 0.001
Learning Problems/Inattention (Median, IQR)	(*N* = 15) 75.5 (56.5–83)	(*N* = 11) 46.0 (44–53.5)	< 0.001
Oppositivity (Median, IQR)	(*N* = 15) 57.5 (47.3–72.8)	(*N* = 11) 44.0 (39.5–53)	< 0.001
*Hand Dominance*	*N* = *5 Left N* = *11 Right*	*N* = *2 Left N* = *14 Right*	0.39

*Note:* Statistical tests used: chi‐squared test (sex) and Mann–Whitney U Test (age, mABC‐2 total score and manual dexterity domain, DCDQ’07, eConner subscales).

Abbreviations: DCD, children with Developmental Coordination Disorder; F, female; IQ, intelligence quotient; IQR, interquartile range; M, male; mABC‐2, Movement ABC‐2 test; TD, typically developing children.

### 3.2 Unimanual and Bimanual Dexterity Outcomes for the Tyneside Pegboard Test

Mean and standard deviations of the different unimanual, bimanual and dual‐task conditions are reported in Table 2.

**Table 2:** Mean and standard deviation of completion time (seconds) for unimanual, bimanual and dual‐task conditions.

	DCD			TD		
	MEAN	SD	MIN‐MAX	MEAN	SD	MIN‐MAX
**UNIMANUAL CONDITIONS**						
LARGE PEGS, DOM L‐R	15.20	2.78	10.75–19.99	12.59	2.76	9.86–19.37
LARGE PEGS, DOM R‐L	15.12	2.85	9.64–20.95	12.80	2.42	9.81–19.41
LARGE PEGS, NON‐DOM L‐R	17.33	3.65	11.82–24.06	14.63	3.36	10.11–22.89
LARGE PEGS, NON‐DOM R‐L	17.30	3.28	12.57–23.54	14.91	3.09	11.06–20.48
SMALL PEGS, DOM L‐R	17.39	5.42	10.76–33.37	14.57	2.75	11.32–22.34
SMALL PEGS, DOM R‐L	16.93	4.49	10.36–29.19	15.42	2.60	11.09–20.34
SMALL PEGS, NON‐DOM L‐R	19.15	4.68	11.42–30.88	16.84	3.26	11.79–23.1
SMALL PEGS, NON‐DOM R‐L	20.03	5.54	12.32–31.54	17.40	3.12	13.09–24.15
**BIMANUAL CONDITION**						
LARGE PEGS (DOM + NON‐DOM)/2	25.61	8.71	13.80–44.68	20.60	5.50	12.89–30.64
**DUAL‐TASK CONDITIONS**						
SMALL PEGS DOM L‐R	16.95	4.60	10.34–27.07	16.51	4.28	12.98–28.28
SMALL PEGS, NON‐DOM L‐R	20.05	4.73	13.35–30.22	15.87	2.65	12.5–21.66

Abbreviations: DCD, developmental coordination disorder; DOM, dominant hand; L‐R, left to right; MAX, maximum value; MIN, minimum value; NON‐DOM; nondominant hand; R‐L, right to left; TD, typically developing.

### 3.3 Unimanual Conditions

Repeated measures ANOVA showed a main group effect in the performance of the TPT tasks (F(1, 30) = 5.55, *p* = 0.03 with a large effect size (partial eta squared = 0.16), which means that children with DCD have a poorer performance overall of unimanual dexterity tasks than TD children.

**Figure 2:**
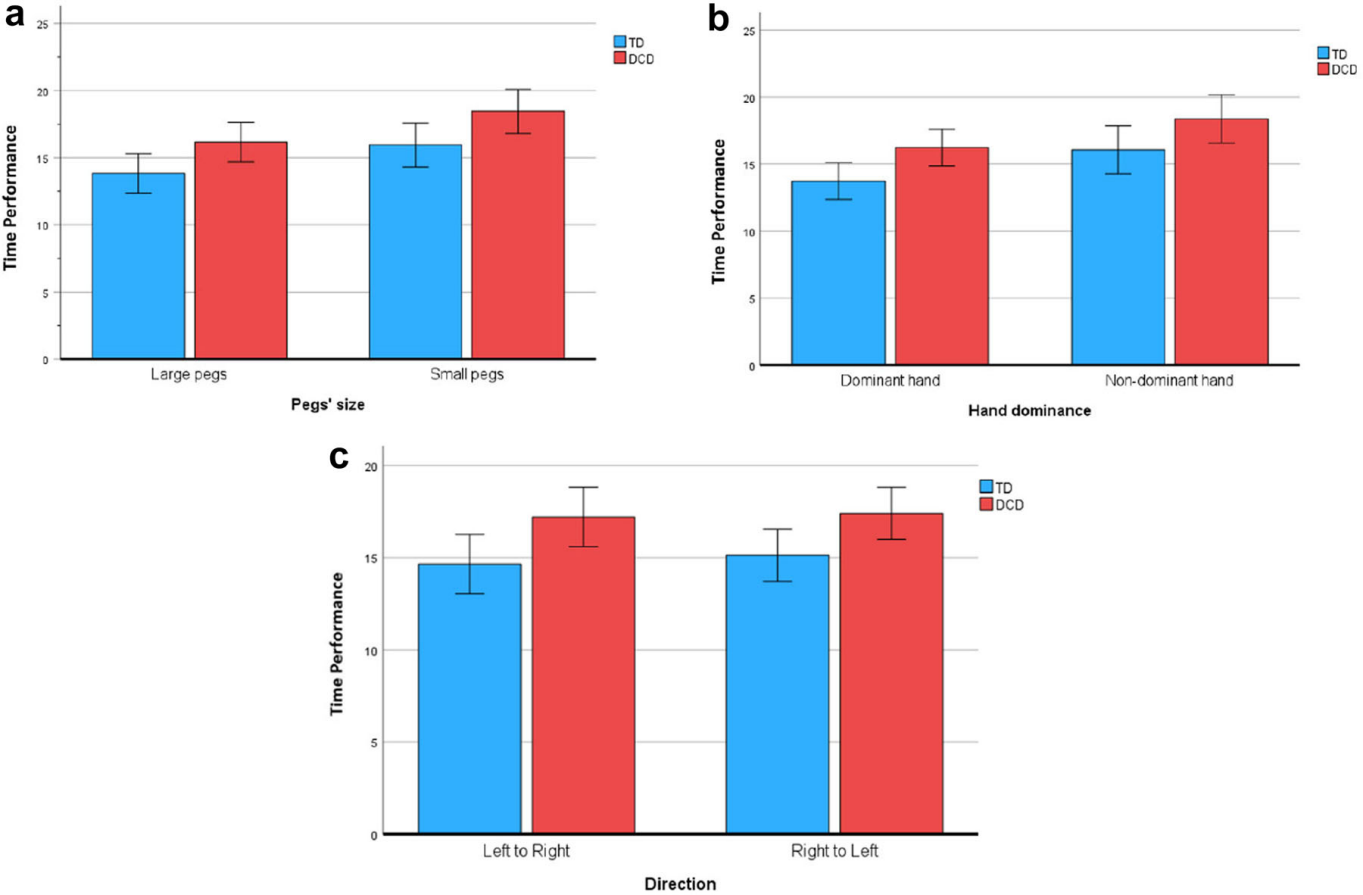
(a, b, c) Comparison of unimanual tasks performed with small and large pegs, dominant and nondominant hand between children with DCD and TD children (2a); with dominant and nondominant hand between children with DCD and TD children (2b) and in both directions between children with DCD and TD children (2c).

A main effect of peg size was found evidencing slower performances with small pegs for both groups compared to performances with large pegs (F(1, 30) = 40.39, *p* < 0.001, with a large effect size (partial eta squared = 0.57) (Figure 2a). However, no impact of the size of pegs between the two groups F(1, 30) = 0.08, *p* = 0.78) was found.

Tasks performed with the nondominant hand were more difficult than with the dominant hand for both groups F(1, 30) = 30.15, *p* < 0.001, with a large effect size (partial eta squared = 0.50) (Figure 2b). There was no interaction effect for hand by group (F(1, 30) = 0.05, *p* = 0.83). Direction of the performance (nondominant side to dominant side and vice versa) had no influence on the performance (F(1, 30) = 2.21, *p* = 0.14) and there was no interaction effect of direction with group (F(1,30) = 2.23, *p* = 0.15) (Figure 2c).

Repeated measures ANOVA showed a main group effect in the performance of the TPT tasks (F(1, 30) = 5.55, *p* = 0.03, with a large effect size, partial eta squared = 0.16), which means that children with DCD have a poorer performance overall of unimanual dexterity tasks than TD children.

A main effect of peg size was found, with both groups performing more slowly with small pegs than with large pegs (F(1, 30) = 40.39, *p* < 0.001, partial eta squared = 0.57) (Figure 2a). However, no impact of the size of pegs between the two groups was found (F(1, 30) = 0.08, *p* = 0.78).

Tasks performed with the nondominant hand were more difficult than than with the dominant hand for both groups (F(1, 30) = 30.15, *p* < 0.001, partial eta squared = 0.50) (Figure 2b). There was no interaction effect for hand by group (F(1, 30) = 0.05, *p* = 0.83). Direction of the performance (nondominant side to dominant side and vice versa) had no influence on the performance F(1, 30) = 2.21, *p* = 0.14), and there was no interaction effect of direction with group (F(1, 30) = 2.23, *p* = 0.15) (Figure 2c).

### 3.4 Bimanual Condition Versus Unimanual Conditions (Large Pegs)

**Figure 3:**
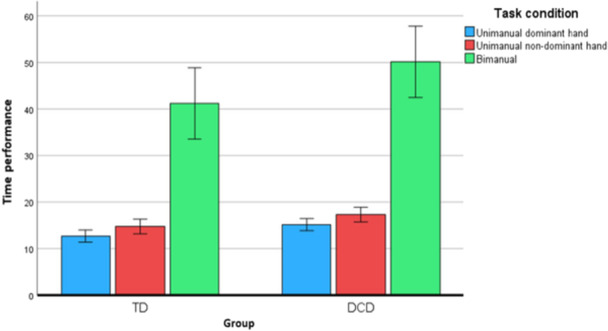
Comparison between children with DCD and TD children of unimanual dominant hand, unimanual nondominant hand and bimanual condition (large pegs).

We found a main effect of condition (F (2, 60) = 57.57, *p* < 0.001) with a large effect size (partial eta squared = 0.66), which means there is a significant difference in performance between unimanual and bimanual conditions. No interaction effect for group by condition was found F(2, 60) = 0.86, *p* = 0.38 (Figure 3), implying that the difference in performance between the three conditions was similar for both groups.

### 3.5 Effect of Dual‐Tasking

#### 3.5.1 Single Task Versus Dual‐Task

**Figure 4:**
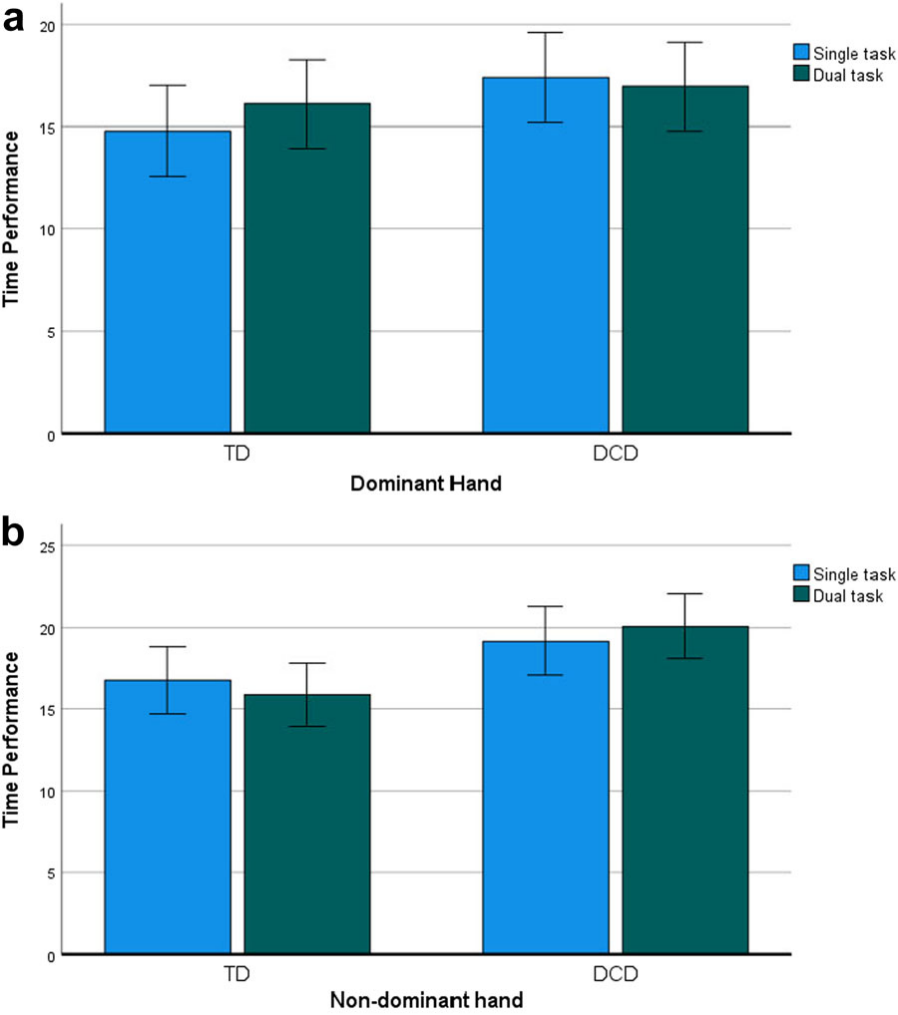
(a) Comparison of performance with dominant hand in single and dual task condition for both groups. (b) Comparison of performance with nondominant hand in single and dual task condition in both groups.

Table 3 summarizes the results of the repeated measures ANOVA. A statistically significant overall group difference in performance across single and dual tasks was found (F(1, 30) = 4.15, *p* = 0.04), indicating that children with DCD were slower than TD children in both task conditions, although no significant effect of task was observed (*p* = 0.67). Additionally, a significant effect of hand dominance was identified (F (1, 30) = 11.80, *p* = 0.002)). A three‐way interaction effect between group, hand, and task (F(1, 30) = 6.29, *p* = 0.02) emerged, highlighting different patterns of performance for the dominant and nondominant hands across task conditions for each group. To further explore the effect of task within groups, separate repeated measures ANOVAs were conducted for each hand. No significant task‐by‐group interactions were observed for either hand (dominant hand: *p* = 0.12; nondominant hand: *p* = 0.19). Moreover, no significant differences were found between single and dual‐task performance for either the dominant hand (F(1, 30) = 0.64, *p* = 0.43) or the nondominant hand (F(1, 30) = 0.00, *p* = 0.99) across both groups. The performance results by hand are illustrated in Figure 4a and 4b.

**Table 3:** Results of the repeated measures ANOVA to assess the effect of dual‐tasking.

Independent variable	*F (df)*	*p*	*η²*
Single task/dual‐task	0.20 (1,30)	0.67	0.01
Group	4.15 (1, 30)	**0.04**	0.13
Hand dominance	11.80 (1,30)	**0.002**	0.28
Task by Group	0.001 (1,30)	0.98	0.00
Task by Hand	0.38 (1,30)	0.55	0.01
Hand by Group	2.64 (1,30)	0.16	0.08
Task by Hand by Group	6.29 (1,30)	**0.02**	0.17

*Note:* Significant results in bold. df, degrees of freedom; η², eta squared.

**Figure 5:**
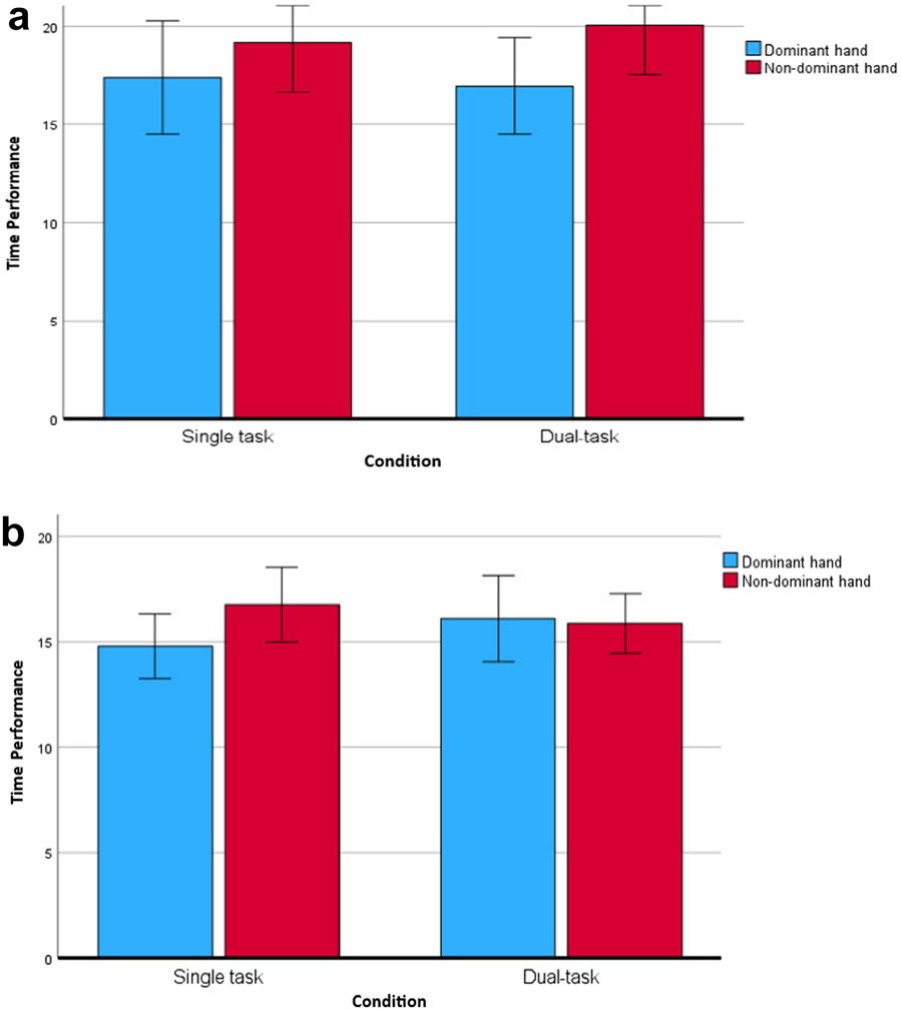
(a) Single and dual‐task performances with dominant and nondominant hand in children with DCD. (b) Single and dual‐task performances with dominant and nondominant hand in TD children.

Further post‐hoc analysis revealed that the interaction effect could be explained by the differential impact of the hand used in both task conditions for each of the two groups. Specifically, post‐hoc analyses of the two groups separately showed that children with DCD demonstrated a significant effect of hand dominance (F(1, 15) = 9.45, *p* < 0.001), with the nondominant hand being slower than the dominant hand in both single and dual‐task conditions (Figure 5a).

In TD children (Figure 5b), a significant interaction between task condition and hand dominance was observed (F(1, 15) = 7.50, *p* = 0.02), indicating differential performance of the dominant and nondominant hands across single‐ and dual‐task conditions. Specifically, during single‐task conditions, the dominant hand was faster compared to the nondominant hand. Conversely, under dual‐task conditions, no significant differences were found between the two hands (*p* = 0.72).

#### 3.5.2 Dual‐Task and eConners' ADHD Index

Results of the correlation analysis were based on 26 participants (11 DCD, 15 TD) because not all parents completed the questionnaires. Attentional difficulties, assessed with the ADHD index of the eConners questionnaire, were examined in relation to performance in the dual‐task condition using both the dominant and the nondominant hand. The partial correlation between ADHD index and the performance with dominant and nondominant hand in dual‐task condition with control of group was not significant (*r* = −0.21, *p* = 0.31; *r* = 0.11, *p* = 0.61 respectively).

## 4 Discussion

The first aim of the present study was to examine unimanual and bimanual dexterity in children with and without DCD. We used the Tyneside Pegboard Test (TPT) under varying task constraints.

Secondary aims were to investigate the automatization deficit hypothesis of motor learning, using a dual motor auditory non‐verbal paradigm, and the impact of attentional difficulties on the dual‐task performance. We found poorer performance by the children with DCD than by TD children in all task conditions. However, the influence of increased task constraints was not larger in children with DCD. Attentional difficulties were not correlated with the performance of dominant or nondominant hand during the dual task when controlling for groups. Below, the results are discussed in more detail.

### 4.1 Single Task Performances

Children with DCD were consistently slower compared to TD children across all unimanual and bimanual TPT conditions, regardless of the hand used, peg size, or movement direction. This general motor slowness aligns with the well‐documented manual dexterity difficulties observed in children with DCD, which can impact school‐related tasks and daily activities (Asonitou et al. 2012; Smits‐Engelsman et al. 2003).

In our study, both children with and without DCD performed more slowly when using the nondominant hand compared to the dominant hand, with no significant group‐by‐hand interaction. This finding aligns with the expected motor asymmetry typically observed in manual tasks, where the dominant hand tends to be more efficient. However, the literature reports contradictory findings regarding dominant versus nondominant hand performance in children with DCD.

For example, Kanj et al. (2022) found a significant difference between children with DCD and their typically developing (TD) peers only in tasks performed with the dominant hand, using the Grooved Pegboard Test. Several factors may explain this discrepancy. Notably, their DCD subgroup was very small (*n* = 8) and embedded within a much larger, highly heterogeneous sample that included individuals with ADHD, intellectual disabilities, and developmental language disorder. Furthermore, the sample spanned an extremely broad age range (5–74 years), which limits the comparability of their findings. Our sample, by contrast, was more homogeneous, consisting of Italian children with a narrow age range (mean age ≈ 7 years, 9 months), and diagnoses limited to DCD or typical development. This difference in sample composition likely contributes to the divergent findings.

Additionally, cultural and linguistic factors may influence manual task performance. Participants in Kanj et al.'s study (2022) were Arabic speakers, accustomed to right‐to‐left literacy, whereas our participants were trained in left‐to‐right script. Previous research suggests that the directionality of reading and writing can impact visuomotor coordination and motor planning, potentially influencing performance in tasks involving spatial sequencing and directional movement. Therefore, literacy direction should be considered a relevant variable when comparing manual dexterity across culturally distinct populations.

Beyond cultural influences, biomechanical and neurodevelopmental factors also play a crucial role in shaping manual performance patterns.

Together, these findings suggest that while hand dominance effects are present across both groups, their expression and detectability may vary depending on the population studied, the tools used, and contextual variables such as cultural background and task design. Our results reinforce the importance of considering both biological and environmental factors when assessing manual performance in children with and without DCD.

Our methodological choice to assess both dominant and nondominant hand performance aligns with the approach of Grohs et al. (2021), who emphasized the importance of evaluating both limbs when investigating motor functioning in children with DCD. Their findings showed more pronounced impairments in the nondominant limb, though deficits were also present in the dominant hand, confirming that motor difficulties in DCD are bilateral. This supports the need for comprehensive bilateral assessment in both research and clinical contexts. Although our bimanual task did not involve simultaneous hand use, both limbs contributed toward a common goal, potentially offering insights into bimanual coordination challenges. Moreover, Grohs et al. (2021) found that children with DCD performed worse than TD peers on a cognitively demanding bimanual task, despite similar hand bias across groups. These findings underscore the relevance of evaluating bimanual performance in tasks requiring interlimb coordination. Future studies with larger samples are needed to clarify motor trajectories in both unimanual and bimanual domains.

Unimanual performance was also influenced by peg size: both groups were slower with smaller pegs, in line with Fitts’ law (Fitts 1954). This confirms previous findings (Ferguson et al. 2015; Smits‐Engelsman et al. 2003) indicating that children with DCD adjust their movements based on task difficulty similarly to TD children, though with generally slower execution.

No differences were observed when crossing the midline in either group, which is consistent with prior studies (Smits‐Engelsman et al. 2006) showing that midline crossing does not impair goal‐directed movement in children with or without DCD.

### 4.2 Bimanual Task Performance

Based on previous findings, bimanual coordination requires not only manual dexterity but also effective action planning (Smits‐Engelsman et al. 2013; Wilson, et al. 2013; Reynolds et al. 2015; Visser et al. 1998). This includes processes such as grasping, orienting the object, and transferring it between hands, along with the simultaneous control of both limbs. Given these demands, it was reasonable to expect that children with DCD would show greater difficulties in the bimanual condition compared to their typically developing peers.

In support of this, earlier research has shown that children with DCD display poorer manual performance, including reduced dexterity, slower reaction times, and greater movement variability during fine motor tasks (Licari et al. 2018; Smits‐Engelsman and Verbecque 2022).

To our knowledge, this was the first study to compare unimanual and bimanual performance in a fine motor task involving object manipulation. As expected, the bimanual condition—requiring the transfer of a peg from one hand to the other before placing it into the pegboard—was more time‐consuming than the unimanual condition across all participants, indicating greater task complexity. However, contrary to our hypothesis, the increase in task demands did not disproportionately affect children with DCD: no significant group differences were found in the relative change performance between the unimanual and bimanual conditions.

This finding is consistent with the results of Grohs et al. (2021), who used unimanual and bimanual tasks to examine motor control in children with and without DCD. In their study, the unimanual task involved visually guided reaching, while the bimanual task consisted of a virtual object‐hitting task using both hands. Similar to our results, they found no significant hand bias or group differences, and concluded that bimanual coordination deficits in DCD may require more targeted assessment.

The lack of group differences in our study may be explained by the nature of the bimanual task employed, which involved a sequential rather than simultaneous coordination pattern. As such, it may not have fully captured the bilateral motor integration difficulties typically observed in children with DCD (Reynolds et al. 2021). These findings underscore the importance of task selection, suggesting that not all bimanual tasks are equally sensitive in detecting motor coordination impairments. Future research should consider tasks with greater temporal coupling, spatial interdependence, or interlimb interference to more effectively assess bimanual motor deficits in this population.

To sum up, we did not find any interaction of hand dominance, peg size, movement direction, unimanual/bimanual task with group, underlining the fact that children with DCD do not present a higher impact of task constraints in the performance of the TPT's unimanual and bimanual tasks compared to TD children. They just exhibit a general slowness in all TPT tasks, which is in line with previous findings (Asonitou et al. 2012; Farhat et al. 2016; Kanj et al. 2022). This result seems coherent with the general slowness hypothesis (Smits‐Engelsman et al. 2003), according to which children with DCD present with an overall slowness in both cognitive and motor tasks compared to peers (Jongmans et al. 2003), since they may need more time to process sensory information and decide which movement to make in a given context. For the children with DCD who also have visual‐perceptual difficulties, a difficulty in sensory processing could lead to a greater use of the visual input, further slowing down the execution of the manual task. Insights on the slowness presented by children with DCD might be taken into consideration in further studies.

### 4.3 Dual‐Task Performance

Our results showed no significant differences between single and dual‐task performance in either group, for both dominant and nondominant hands. This finding does not support the automatization deficit hypothesis (Visser 2003). Children with DCD were consistently slower than their typically developing (TD) peers across all task conditions, which aligns with findings by Michel and Molitor (2022), who reported no dual‐task cost in fine motor performance among children with motor difficulties at kindergarten age.

These results suggest that manual dexterity difficulties in children with DCD may be better explained by alternative models, such as the internal modelling deficit hypothesis. Further research should investigate the automatization deficit hypothesis using more complex and ecologically valid fine motor tasks, such as handwriting. For example, tasks like writing one's name in capital letters may provide a more demanding context in which automatization limitations could emerge, though the involvement of additional cognitive and linguistic skills must be considered. Similarly, experimental tasks contrasting complex rhythmic versus arrhythmic motor sequences—such as those proposed by Biotteau et al. (2015)—may prove useful in further exploring this hypothesis.

In the single‐task condition, TD children exhibited a performance pattern similar to that of the DCD group, with slower execution using the nondominant hand. However, under the dual‐task condition, their behaviour diverged: unlike the DCD group, TD children maintained or even improved their performance with the nondominant hand. As suggested by Schaefer et al. (2008), this may reflect an adaptive mechanism whereby increased task difficulty prompts a compensatory shift of attentional resources toward the motor task, allowing TD children to preserve or enhance performance despite the additional cognitive load

Given that the nondominant hand typically requires more attention for precise movements, we hypothesized a correlation between dual‐task performance with the nondominant hand and attentional difficulties, as measured by the ADHD index. However, this was not supported by our data. Despite the DCD group scoring significantly higher on the eConners ADHD index, no significant correlation was found between attentional scores and performance under cognitive load. This aligns with several previous studies. For instance, Miyahara et al. (2006) found no significant differences in manual coordination between children with DCD alone and those with co‐occurring DCD and ADHD, suggesting that attention deficits were not the primary driver of poor motor performance. Similarly, Pitcher et al. (2003) observed that children with ADHD only did not differ from controls in fine motor ability, whereas those with both ADHD and DCD showed significantly poorer performance—again implicating motor‐specific rather than attentional causes.

These findings are further supported by research on procedural learning using the Serial Reaction Time Task (SRTT). Studies by Gheysen et al. (2011), Lejeune et al. (2013), and more recently Wilson et al. (2020), indicate that individuals with DCD show reduced procedural learning not explained by attentional deficits. Even in adult populations (e.g., Sinani et al. 2024), individuals with DCD do not exhibit attention‐related impairments during procedural motor tasks. These results suggest that the difficulties seen in DCD reflect a core deficit in motor learning mechanisms rather than limitations in attention.

In summary, our findings suggest that while typically developing (TD) children may adapt to increased task demands—possibly through attentional reallocation—children with DCD exhibit consistently reduced performance across manual tasks, regardless of cognitive load. This pattern indicates that their difficulties are likely rooted in motor coordination mechanisms rather than attentional control. Although the dual‐task condition did not reveal additional performance decrements in the DCD group, this does not support the automatization deficit hypothesis in the present context. Instead, our results align more closely with alternative models such as the internal modelling deficit hypothesis.

Moreover, the lack of a significant relationship between dual‐task performance and attentional scores further reinforces the view that attentional difficulties are not the primary drivers of motor impairment in DCD. These conclusions are consistent with previous findings on procedural learning and fine motor control in both pediatric and adult DCD populations.

Therefore, interventions targeting children with DCD should prioritize motor‐specific training, particularly approaches that challenge coordination under realistic and cognitively demanding conditions. Future research should employ ecologically valid tasks—such as handwriting or rhythmically complex actions—to more effectively test the automatization hypothesis and further elucidate the underlying mechanisms of motor impairment in DCD.

### 4.4 Strengths and Limitations

The present study does have some limitations. Since joint hypermobility and muscle hypotonia represent common clinical features which may impact speed and accuracy (Gibbs et al. 2007) we could have considered their potential impact on unimanual and bimanual dexterity performances. However, the assessment of joint hypermobility and muscle hypotonia were not part of the protocol. Secondly, we did not assess cognitive task performance in a single‐task condition, which prevented us from calculating the Dual Task Effect (DTE). The DTE is widely recognized as a more comprehensive indicator of dual‐task interference, as it accounts for changes in both cognitive and motor performance (Plummer and Eskes 2015). The DTE is particularly important when evaluating dual‐task performance before and after rehabilitation, as it provides insights into the effects of treatment. Although our study was not an intervention study, calculating the DTE would have strengthened our findings. Future studies should consider including this measure, starting with a revision of the task protocol length. In our study, we excluded the cognitive single‐task condition because participants became too fatigued and disengaged after completing all unimanual and bimanual motor tasks.

Third, though the TPT is an electronic device, we decided to use a stopwatch because of the specific protocol used. Furthermore, though the TPT provides normative data, we could not use these as they were not based on our conditions. However, we included an age‐matched control group. Also, the construct validity of this test for use in children with DCD should be further investigated. Fourthly, the sample size in our study was limited, however, COVID‐19 regulations hindered the recruitment of larger samples and samples with more equal sex distribution. Fairbairn et al. (2020), reported that girls at risk of or with probable DCD have significantly greater gross motor and non‐motor difficulties, with significantly greater impact on activities and participation. In contrast, boys at risk of or with probable DCD are reported to have significantly greater challenges related to fine motor skills (Cleaton et al. 2021). Also, for generalization of our findings, one should take into account the recruitment of children with DCD via IRCCS Fondazione Stella Maris, implying a more severe diagnosis and high risk of comorbidities. Furthermore, a considerable proportion of children in the DCD group presented elevated scores on the ADHD Index of the eConners questionnaire, suggesting clinically significant attentional difficulties. While this reflects the real‐world complexity of overlapping neurodevelopmental disorders, it limits our ability to isolate the effects of DCD alone. Due to the small sample size, rerunning the analyses without these participants was not statistically feasible, however, this issue should be addressed in future studies with larger and more heterogeneous samples.

Finally, although sex‐related differences in motor and non‐motor skills have been reported in children with DCD (Fairbairn et al. 2020; Cleaton et al. 2021), our sample included a small and uneven number of males and females across groups (e.g., only four females in the DCD group). As a result, it was not statistically appropriate to include sex as a factor in the repeated measures analyses. Future studies should address this limitation by recruiting larger and more balanced samples to explore potential gender effects more thoroughly.

## 5 Conclusion

This is the first study to offer insights on manual function in children with DCD using different task constraints within a single test protocol, using standardized pegboard conditions. It also offers a comparison of unimanual versus bimanual performance and single versus dual task. Furthermore, interference of attentional difficulties during dual‐task performance was also taken into account.

The results of this study confirm that children with DCD exhibit an overall slowness in unimanual and bimanual fine motor tasks, compared with TD children, not influenced by different task constraints. Slowness remains the main clinical characteristic of dual‐task performance in children with DCD. The hypothesis that manual dexterity difficulties expressed by children with DCD might be explained by the automatization deficit hypothesis is not confirmed in our study.

Further research is needed to investigate the automatization deficit hypothesis in more complex fine motor tasks, with larger and more representative samples of children with DCD and age‐ and sex‐matched TD children. Cognitive tasks in both single‐ and dual‐ task conditions should also be investigated, to allow calculation of the dual‐task effect.

Attentional difficulties, particularly in cognitively demanding tasks, may further impair motor performance. Therefore, integrated approaches addressing both motor and cognitive components may be especially beneficial, particularly in cases of DCD with co‐occurring ADHD. Clinical assessments should consider both motor and cognitive aspects to develop individualized and effective intervention plans.

## Conflicts of Interest

The authors declare no conflicts of interest.

## Supporting information

Appendix A revised.

Appendix B supplementary material.

## Data Availability

The data that support the findings of this study are available on request from the corresponding author. The data are not publicly available due to privacy or ethical restrictions.
