# Supplementary material for: Manual Dexterity Abilities and Dual Tasking in Children With Developmental Coordination Disorder and Typically Developing Children
Source: J Clin Psychol. 2025 Oct 21;82(2):146–60. doi: 10.1002/jclp.70051 (PMC12793828; doi:10.1002/jclp.70051)
Supplement: Supplementary file 1 — Appendix A revised. [file JCLP-82-146-s001.docx]

**PEGBOARD CHECKLIST (Sheet A)**

| ID - Name | Date of birth | Date | Name tester |
| --- | --- | --- | --- |
|  |  |  |  |

|  | **Trial** | **Direction** | **Hand** | **Procedural instructions** |
| --- | --- | --- | --- | --- |
| **UNIMANUAL CONDITIONS**  **WITH LARGE PEGS** |  | L > R | Practice trial with 3 pegs | - Starting position: from left to right - Hand: participants start with their dominant or non-dominant hand according to the assigned protocol - Prompt verbal message: “*Move the pegs from one board to the other as fast as you can.*” |
|  | 1 | L > R | Dominant hand |  |
|  | 2 | R > L | Dominant hand |  |
|  | 3 | L > R | Dominant hand |  |
|  | 4 | R > L | Dominant hand |  |
|  | 5 | L > R | Non-dominant hand |  |
|  | 6 | R > L | Non-dominant hand |  |
|  | 7 | L > R | Non-dominant hand |  |
|  | 8 | R > L | Non-dominant hand |  |
| **BIMANUAL CONDITIONS**  **WITH LARGE PEGS** |  | L > R | Practice trial with 3 pegs | - Starting position: from left to right - Hand: participants start with their left hand - Prompt verbal message “*Move the pegs from one board to the other by passing them through the hole. Once you have passed the peg through the hole with one hand, take the peg with the other hand and insert it into the hole. Do not try to insert the peg with the same hand.”* |
|  | 9 | L > R | Left hand leading |  |
|  | 10 | R > L | Right hand leading |  |
| **UNIMANUAL CONDITIONS**  **WITH SMALL PEGS** |  | L > R | Practice trial with 3 pegs | - Starting position: from left to right - Hand: participants start with their dominant or non-dominant hand according to the assigned protocol - Prompt verbal message: “Move the pegs from one board to the other as fast as you can.” |
|  | 11 | L > R | Dominant hand |  |
|  | 12 | R > L | Dominant hand |  |
|  | 13 | L > R | Dominant hand |  |
|  | 14 | R > L | Dominant hand |  |
|  | 15 | L > R | Non-dominant hand |  |
|  | 16 | R > L | Non-dominant hand |  |
|  | 17 | L > R | Non-dominant hand |  |
|  | 18 | R > L | Non-dominant hand |  |
| **DUAL TASK CONDITIONS UNIMANUAL SMALL PEGS + ACOUSTIC TASK** |  | L > R | Practice trial with 3 pegs | - Starting position: from left to right - Participants start with the dominant or non-dominant hand according to the assigned protocol Prompt verbal message *“Move the pegs from one board to the other as quickly as you can, just as you did before, while listening to the sounds we have practised. When you hear the sound of a helicopter, please say “yes”. When you hear the sound of an airplane say nothing.”* |
|  | 19 | L > R | Dominant hand |  |
|  | 20 | L > R | Dominant hand |  |
|  | 21 | R > L | Non-dominant hand |  |
|  | 22 | R > L | Non-dominant hand |  |

**PEGBOARD CHECKLIST (Sheet B)**

| ID - Name | Date of birth | Date | Name tester |
| --- | --- | --- | --- |
|  |  |  |  |

|  | **Trial** | **Direction** | **Hand** | **Procedural instructions** |
| --- | --- | --- | --- | --- |
| **UNIMANUAL CONDITIONS**  **WITH LARGE PEGS** |  | L > R | Practice trial with 3 pegs | - Starting position: from left to right - Hand: participants start with their dominant or non-dominant hand according to the assigned protocol - Prompt verbal message: “*Move the pegs from one board to the other as fast as you can.*” |
|  | 1 | L > R | Non-dominant hand |  |
|  | 2 | R > L | Non-dominant hand |  |
|  | 3 | L > R | Non-dominant hand |  |
|  | 4 | R > L | Non-dominant hand |  |
|  | 5 | L > R | Dominant hand |  |
|  | 6 | R > L | Dominant hand |  |
|  | 7 | L > R | Dominant hand |  |
|  | 8 | R > L | Dominant hand |  |
| **BIMANUAL CONDITIONS**  **WITH LARGE PEGS** |  | L > R | Practice trial with 3 pegs | - Starting position: from left to right - Hand: participants start with their left hand - Prompt verbal message “*Move the pegs from one board to the other by passing them through the hole. Once you have passed the peg through the hole with one hand, take the peg with the other hand and insert it into the hole. Do not try to insert the peg with the same hand.”* |
|  | 9 | L > R | Left hand leading |  |
|  | 10 | R > L | Right hand leading |  |
| **UNIMANUAL CONDITIONS**  **WITH SMALL PEGS** |  | L > R | Practice trial with 3 pegs | - Starting position: from left to right - Hand: participants start with their dominant or non-dominant hand according to the assigned protocol - Prompt verbal message: “Move the pegs from one board to the other as fast as you can.” |
|  | 11 | L > R | Non-dominant hand |  |
|  | 12 | R > L | Non-dominant hand |  |
|  | 13 | L > R | Non-dominant hand |  |
|  | 14 | R > L | Non-dominant hand |  |
|  | 15 | L > R | Dominant hand |  |
|  | 16 | R > L | Dominant hand |  |
|  | 17 | L > R | Dominant hand |  |
|  | 18 | R > L | Dominant hand |  |
| **DUAL TASK CONDITIONS UNIMANUAL SMALL PEGS + ACOUSTIC TASK** |  | L > R | Practice trial with 3 pegs | - Starting position: from left to right - Participants start with the dominant or non-dominant hand according to the assigned protocol Prompt verbal message *“Move the pegs from one board to the other as quickly as you can, just as you did before, while listening to the sounds we have practised. When you hear the sound of a helicopter, please say “yes”. When you hear the sound of an airplane say nothing.”* |
|  | 19 | L > R | Non-dominant hand |  |
|  | 20 | L > R | Non-dominant hand |  |
|  | 21 | R > L | Dominant hand |  |
|  | 22 | R > L | Dominant hand |  |

**PEGBOARD CHECKLIST (Sheet C)**

| ID - Name | Date of birth | Date | Name tester |
| --- | --- | --- | --- |
|  |  |  |  |

|  | **Trial** | **Direction** | **Hand** | **Procedural instructions** |
| --- | --- | --- | --- | --- |
| **UNIMANUAL CONDITIONS**  **WITH LARGE PEGS** |  | L > R | Practice trial with 3 pegs | - Starting position: from left to right - Hand: participants start with their dominant or non-dominant hand according to the assigned protocol - Prompt verbal message: “*Move the pegs from one board to the other as fast as you can.*” |
|  | 1 | L > R | Dominant hand |  |
|  | 2 | R > L | Dominant hand |  |
|  | 3 | L > R | Dominant hand |  |
|  | 4 | R > L | Dominant hand |  |
|  | 5 | L > R | Non-dominant hand |  |
|  | 6 | R > L | Non-dominant hand |  |
|  | 7 | L > R | Non-dominant hand |  |
|  | 8 | R > L | Non-dominant hand |  |
| **BIMANUAL CONDITIONS**  **WITH LARGE PEGS** |  | L > R | Practice trial with 3 pegs | - Starting position: from left to right - Hand: participants start with their left hand - Prompt verbal message “*Move the pegs from one board to the other by passing them through the hole. Once you have passed the peg through the hole with one hand, take the peg with the other hand and insert it into the hole. Do not try to insert the peg with the same hand.”* |
|  | 9 | L > R | Left hand leading |  |
|  | 10 | R > L | Right hand leading |  |
| **UNIMANUAL CONDITIONS**  **WITH SMALL PEGS** |  | L > R | Practice trial with 3 pegs | - Starting position: from left to right - Hand: participants start with their dominant or non-dominant hand according to the assigned protocol - Prompt verbal message: “Move the pegs from one board to the other as fast as you can.” |
|  | 11 | L > R | Non-dominant hand |  |
|  | 12 | R > L | Non-dominant hand |  |
|  | 13 | L > R | Non-dominant hand |  |
|  | 14 | R > L | Non-dominant hand |  |
|  | 15 | L > R | Dominant hand |  |
|  | 16 | R > L | Dominant hand |  |
|  | 17 | L > R | Dominant hand |  |
|  | 18 | R > L | Dominant hand |  |
| **DUAL TASK CONDITIONS UNIMANUAL SMALL PEGS + ACOUSTIC TASK** |  | L > R | Practice trial with 3 pegs | - Starting position: from left to right - Participants start with the dominant or non-dominant hand according to the assigned protocol Prompt verbal message *“Move the pegs from one board to the other as quickly as you can, just as you did before, while listening to the sounds we have practised. When you hear the sound of a helicopter, please say “yes”. When you hear the sound of an airplane say nothing.”* |
|  | 19 | L > R | Dominant hand |  |
|  | 20 | L > R | Dominant hand |  |
|  | 21 | R > L | Non-dominant hand |  |
|  | 22 | R > L | Non-dominant hand |  |

**PEGBOARD CHECKLIST (Sheet D)**

| ID - Name | Date of birth | Date | Name tester |
| --- | --- | --- | --- |
|  |  |  |  |

|  | **Trial** | **Direction** | **Hand** | **Procedural instructions** |
| --- | --- | --- | --- | --- |
| **UNIMANUAL CONDITIONS**  **WITH LARGE PEGS** |  | L > R | Practice trial with 3 pegs | - Starting position: from left to right - Hand: participants start with their dominant or non-dominant hand according to the assigned protocol - Prompt verbal message: “*Move the pegs from one board to the other as fast as you can.*” |
|  | 1 | L > R | Dominant hand |  |
|  | 2 | R > L | Dominant hand |  |
|  | 3 | L > R | Dominant hand |  |
|  | 4 | R > L | Dominant hand |  |
|  | 5 | L > R | Non-dominant hand |  |
|  | 6 | R > L | Non-dominant hand |  |
|  | 7 | L > R | Non-dominant hand |  |
|  | 8 | R > L | Non-dominant hand |  |
| **BIMANUAL CONDITIONS**  **WITH LARGE PEGS** |  | L > R | Practice trial with 3 pegs | - Starting position: from left to right - Hand: participants start with their left hand - Prompt verbal message “*Move the pegs from one board to the other by passing them through the hole. Once you have passed the peg through the hole with one hand, take the peg with the other hand and insert it into the hole. Do not try to insert the peg with the same hand.”* |
|  | 9 | L > R | Left hand leading |  |
|  | 10 | R > L | Right hand leading |  |
| **UNIMANUAL CONDITIONS**  **WITH SMALL PEGS** |  | L > R | Practice trial with 3 pegs | - Starting position: from left to right - Hand: participants start with their dominant or non-dominant hand according to the assigned protocol - Prompt verbal message: “Move the pegs from one board to the other as fast as you can.” |
|  | 11 | L > R | Non-dominant hand |  |
|  | 12 | R > L | Non-dominant hand |  |
|  | 13 | L > R | Non-dominant hand |  |
|  | 14 | R > L | Non-dominant hand |  |
|  | 15 | L > R | Dominant hand |  |
|  | 16 | R > L | Dominant hand |  |
|  | 17 | L > R | Dominant hand |  |
|  | 18 | R > L | Dominant hand |  |
| **DUAL TASK CONDITIONS UNIMANUAL SMALL PEGS + ACOUSTIC TASK** |  | L > R |  | - Starting position: from left to right - Participants start with the dominant or non-dominant hand according to the assigned protocol Prompt verbal message *“Move the pegs from one board to the other as quickly as you can, just as you did before, while listening to the sounds we have practised. When you hear the sound of a helicopter, please say “yes”. When you hear the sound of an airplane say nothing.”* |
|  | 19 | L > R | Non-dominant hand |  |
|  | 20 | L > R | Non-dominant hand |  |
|  | 21 | R > L | Dominant hand |  |
|  | 22 | R > L | Dominant hand |  |

**PEGBOARD CHECKLIST (Sheet E)**

| ID - Name | Date of birth | Date | Name tester |
| --- | --- | --- | --- |
|  |  |  |  |

|  | **Trial** | **Direction** | **Hand** | **Procedural instructions** |
| --- | --- | --- | --- | --- |
| **UNIMANUAL CONDITIONS**  **WITH LARGE PEGS** |  | L > R | Practice trial with 3 pegs | - Starting position: from left to right - Hand: participants start with their dominant or non-dominant hand according to the assigned protocol - Prompt verbal message: “*Move the pegs from one board to the other as fast as you can.*” |
|  | 1 | L > R | Non-dominant hand |  |
|  | 2 | R > L | Non-dominant hand |  |
|  | 3 | L > R | Non-dominant hand |  |
|  | 4 | R > L | Non-dominant hand |  |
|  | 5 | L > R | Dominant hand |  |
|  | 6 | R > L | Dominant hand |  |
|  | 7 | L > R | Dominant hand |  |
|  | 8 | R > L | Dominant hand |  |
| **BIMANUAL CONDITIONS**  **WITH LARGE PEGS** |  | L > R | Practice trial with 3 pegs | - Starting position: from left to right - Hand: participants start with their left hand - Prompt verbal message “*Move the pegs from one board to the other by passing them through the hole. Once you have passed the peg through the hole with one hand, take the peg with the other hand and insert it into the hole. Do not try to insert the peg with the same hand.”* |
|  | 9 | L > R | Left hand leading |  |
|  | 10 | R > L | Right hand leading |  |
| **UNIMANUAL CONDITIONS**  **WITH SMALL PEGS** |  | L > R | Practice trial with 3 pegs | - Starting position: from left to right - Hand: participants start with their dominant or non-dominant hand according to the assigned protocol - Prompt verbal message: “Move the pegs from one board to the other as fast as you can.” |
|  | 11 | L > R | Dominant hand |  |
|  | 12 | R > L | Dominant hand |  |
|  | 13 | L > R | Dominant hand |  |
|  | 14 | R > L | Dominant hand |  |
|  | 15 | L > R | Non-dominant hand |  |
|  | 16 | R > L | Non-dominant hand |  |
|  | 17 | L > R | Non-dominant hand |  |
|  | 18 | R > L | Non-dominant hand |  |
| **DUAL TASK CONDITIONS UNIMANUAL SMALL PEGS + ACOUSTIC TASK** |  | L > R |  | - Starting position: from left to right - Participants start with the dominant or non-dominant hand according to the assigned protocol Prompt verbal message *“Move the pegs from one board to the other as quickly as you can, just as you did before, while listening to the sounds we have practised. When you hear the sound of a helicopter, please say “yes”. When you hear the sound of an airplane say nothing.”* |
|  | 19 | L > R | Dominant hand |  |
|  | 20 | L > R | Dominant hand |  |
|  | 21 | R > L | Non-dominant hand |  |
|  | 22 | R > L | Non-dominant hand |  |

**PEGBOARD CHECKLIST (Sheet F)**

| ID – Name | Date of birth | Date | Name tester |
| --- | --- | --- | --- |
|  |  |  |  |

|  | **Trial** | **Direction** | **Hand** | **Procedural instructions** |
| --- | --- | --- | --- | --- |
| **UNIMANUAL CONDITIONS**  **WITH LARGE PEGS** |  | L > R | Practice trial with 3 pegs | - Starting position: from left to right - Hand: participants start with their dominant or non-dominant hand according to the assigned protocol - Prompt verbal message: “*Move the pegs from one board to the other as fast as you can.*” |
|  | 1 | L > R | Non-dominant hand |  |
|  | 2 | R > L | Non-dominant hand |  |
|  | 3 | L > R | Non-dominant hand |  |
|  | 4 | R > L | Non-dominant hand |  |
|  | 5 | L > R | Dominant hand |  |
|  | 6 | R > L | Dominant hand |  |
|  | 7 | L > R | Dominant hand |  |
|  | 8 | R > L | Dominant hand |  |
| **BIMANUAL CONDITIONS**  **WITH LARGE PEGS** |  | L > R | Practice trial with 3 pegs | - Starting position: from left to right - Hand: participants start with their left hand - Prompt verbal message “*Move the pegs from one board to the other by passing them through the hole. Once you have passed the peg through the hole with one hand, take the peg with the other hand and insert it into the hole. Do not try to insert the peg with the same hand.”* |
|  | 9 | L > R | Left hand leading |  |
|  | 10 | R > L | Right hand leading |  |
| **UNIMANUAL CONDITIONS**  **WITH SMALL PEGS** |  | L > R | Practice trial with 3 pegs | - Starting position: from left to right - Hand: participants start with their dominant or non-dominant hand according to the assigned protocol - Prompt verbal message: “Move the pegs from one board to the other as fast as you can.” |
|  | 11 | L > R | Dominant hand |  |
|  | 12 | R > L | Dominant hand |  |
|  | 13 | L > R | Dominant hand |  |
|  | 14 | R > L | Dominant hand |  |
|  | 15 | L > R | Non-dominant hand |  |
|  | 16 | R > L | Non-dominant hand |  |
|  | 17 | L > R | Non-dominant hand |  |
|  | 18 | R > L | Non-dominant hand |  |
| **DUAL TASK CONDITIONS UNIMANUAL SMALL PEGS + ACOUSTIC TASK** |  | L > R | Practice trial with 3 pegs | - Starting position: from left to right   Participants start with the dominant or non-dominant hand according to the assigned protocol Prompt verbal message *“Move the pegs from one board to the other as quickly as you can, just as you did before, while listening to the sounds we have practised. When you hear the sound of a helicopter, please say “yes”. When you hear the sound of an airplane say nothing.”* |
|  | 19 | L > R | Non-dominant hand |  |
|  | 20 | L > R | Non-dominant hand |  |
|  | 21 | R > L | Dominant hand |  |
|  | 22 | R > L | Dominant hand |  |
