# Supplementary material for: Manual Dexterity Abilities and Dual Tasking in Children With Developmental Coordination Disorder and Typically Developing Children
Source: J Clin Psychol. 2025 Oct 21;82(2):146–60. doi: 10.1002/jclp.70051 (PMC12793828; doi:10.1002/jclp.70051)
Supplement: Supplementary file 2 — Appendix B supplementary material. [file JCLP-82-146-s002.docx]

***Table 1a****.*

*Participants distribution for each subscale of the eConners questionnaire.*

| *eConners subscales* | *Oppositivity* | | *Learning Problems / Inattention* | | *Hyperactivity* | | *ADHD’s index* | |
| --- | --- | --- | --- | --- | --- | --- | --- | --- |
|  | *DCD* | *TD* | *DCD* | *TD* | *DCD* | *TD* | *DCD* | *TD* |
| *Very elevated*  *>70+* | *N= 4* | *N= 0* | *N= 7* | *N= 0* | *N= 5* | *N= 0* | *N= 7* | *N= 0* |
| *Elevated*  *65-69* | *N= 0* | *N= 0* | *N= 1* | *N= 0* | *N= 2* | *N= 0* | *N= 1* | *N= 0* |
| *High average*  *60-64* | *N= 1* | *N= 0* | *N= 0* | *N= 2* | *N= 1* | *N= 1* | *N= 3* | *N= 1* |
| *Average*  *40-59* | *N= 6* | *N= 15* | *N= 3* | *N= 13* | *N= 3* | *N= 14* | *N= 0* | *N= 14* |

*DCD: children with Developmental Coordination Disorder; TD: typically developing children; N= number of patients*
